# Prevalence of Hepatitis C Virus in Aligarh: A Seven Year Experience

**DOI:** 10.4103/0970-0218.55299

**Published:** 2009-07

**Authors:** Farhan Asif Siddiqui, Kafil Akhtar, Rana K Sherwani, Khaliqur Rehman, Feroz Alam, Athar Ansari

**Affiliations:** The Department of Pathology and Community Medicine, JN Medical College, Aligarh Muslim University, Aligarh, India. E-mail: drkafilakhtar@gmail.com

Sir,

Blood transfusion is life saving, but the chances of transmission of microorganisms remain a potential danger. With proper selection of donors on the basis of detailed clinical history and examination, along with the availability of a sophisticated screening procedure, the chances of transmission of diseases through transfusion have reduced considerably. However, the risk is only minimized and not ruled out.

Hepatitis C virus (HCV), the main etiological agent of the clinical entity, formerly known as Non-A, Non-B Hepatitis, was discovered in 1989 by Choo *et al*.(1) HCV along with Hepatitis B virus (HBV) is responsible for the majority of post-transfusion hepatitis.

The present study was done to determine the prevalence rate of HCV antibodies in replacement and voluntary donors attending the Blood Bank of the Jawaharlal Nehru Medical College Hospital, A.M.U., Aligarh, over a period of seven years, from January 2001 to December 2007. Out of 68173 donors, there were 44984 replacement donors and 23189 voluntary donors. No honorary or professional donor was bled. Thereafter, 5 – 10 ml of blood was withdrawn with a 10 ml syringe and subjected to anti-HCV testing, using a commercially available third generation anti-HCV ELISA kit.

A majority of the donors were young males in the age group of 30 – 40 years (60%), followed by 30% donors in the age group of 21 – 30 years.

HCV positive cases among the apparently healthy blood donors were assessed. In the year 2001; there was no donor who showed positivity for HCV antibody. In the year 2002, there was only one positive case per 8130 donations (0.01%). The year 2003 showed an increase in the number of positive cases for HCV, three positive cases per 9577 donations (0.03%). The year 2004 showed a further increase, with eight positive cases per 10842 donations (0.07%). The scenario in 2005 was somewhat similar to that seen in 2003, with five positive cases per 10990 donations (0.04%). There was a steep rise in 2006, with 13 positive cases per 11068 donations (0.12%). The number of positive cases continued to rise in 2007, with 18 positive cases per 11566 donations (0.15%).

A majority of the positive cases belonged to the age group 21 – 30 years, 22 in total (45.83%), followed by 14 cases (29.16%) in the age group 31 – 40 years. There were seven cases (14.58%) in the second decade, while only five positive cases (10.41%) were seen in the fifth decade.

The study revealed only three female positive cases out of a total of 48 positive cases (6.25%) during the entire period of study. One reason for this data could be the overall low number of female donors as compared to male donors.

Transfusion-associated infections continue to be a big threat to the safety of blood supply, moreso in the developing and underdeveloped countries. Viral infections are the major cause of morbidity and mortality in blood recipients and a majority of transfusion-associated hepatitis is caused by the Hepatitis C virus. With approximately 170 million people worldwide estimated to be infected with HCV, a figure that is four times the HIV infection status, it has the potential to be the next pandemic.(2)

The present study was undertaken to determine the prevalence of HCV antibodies in healthy blood donors. The presence of anti-HCV does not constitute a diagnosis of Hepatitis C, but may be indicative of recent and/or past infection by hepatitis C virus. A nonreactive test result does not exclude the possibility of exposure to HCV. Levels of anti-HCV may be undetectable in early infections due to a low titer; polymerase chain reaction (PCR) may play an important role under such circumstances. Even then, screening blood donors for anti-HCV has reduced the incidence of post-transfusion non-A and non-B hepatitis dramatically.

Hepatitis C virus (HCV) is a hepatotropic virus of family Flaviviridae and genus Hepacivirus, having a single standard RNA of positive polarity as genomic material. A large number of genotypes have been identified among hepatitis C virus isolates from all over the world. Presently six main groups of sequence variants have been characterized, corresponding to types 1 – 6; each group containing a number of more closely related subtypes (a, b, c, etc.).(3) Genotype 3 is the most prevalent genotype in patients with chronic hepatitis C in North and Central India, and is associated with significant hepatic steatosis and fibrosis.(4)

The safety of blood transfusion is compromised in India due to its dependence on replacement donors, endemic hepatitis in this region, high cost of screening, and a lack of funds and trained personnel. Williams *et al*., in 1992, conducted a study and showed a seroprevalence of 11.1% for antibodies to HCV in multiple transfused thalassemia major patients.(5) This high prevalence could have been because of improper clinical examination and screening of the donors. However, proper pre-donation screening of donors and rejecting volunteers with a past history of viral hepatitis has shown a lower prevalence of post-transfusion Hepatitis C infection (0.07%) in our study, as compared to a higher prevalence of 2.0% reported by Kothari *et al*.(6)

Our study showed an alarming percentage of HCV positive cases in the younger generation, 36 cases (74.9%), with an upward trend of positivity, with time.

Routine HCV antibody screening and proper clinical examination should be carried out in blood donors with a thrust toward youth surveillance, which will aid in delivery of safe blood.
